# Microalgae *Aurantiochytrium* Sp. Increases Neurogenesis and Improves Spatial Learning and Memory in Senescence-Accelerated Mouse-Prone 8 Mice

**DOI:** 10.3389/fcell.2020.600575

**Published:** 2021-02-09

**Authors:** Kazunori Sasaki, Noelia Geribaldi-Doldán, Qingqing Wu, Julie Davies, Francis G. Szele, Hiroko Isoda

**Affiliations:** ^1^Alliance for Research on the Mediterranean and North Africa (ARENA), University of Tsukuba, Tsukuba, Japan; ^2^Open Innovation Laboratory for Food and Medicinal Resource Engineering, National Institute of Advanced Industrial Science and Technology (AIST), University of Tsukuba, Tsukuba, Japan; ^3^Faculty of Pure and Applied Sciences, University of Tsukuba, Tsukuba, Japan; ^4^Department of Physiology, Anatomy and Genetics, University of Oxford, Oxford, United Kingdom; ^5^Faculty of Life and Environmental Sciences, University of Tsukuba, Tsukuba, Japan

**Keywords:** *Aurantiochytrium* sp., neuroprotection, adult neurogenesis, neuronal stem cells, SAMP8 mice

## Abstract

Much attention has recently been focused on nutraceuticals, with minimal adverse effects, developed for preventing or treating neurological diseases such as Alzheimer's disease (AD). The present study was conducted to investigate the potential effect on neural development and function of the microalgae *Aurantiochytrium* sp. as a nutraceutical. To test neuroprotection by the ethanol extract of *Aurantiochytrium* (EEA) and a derivative, the n-Hexane layer of EEA (HEEA), amyloid-β-stimulated SH-SY5Y cells, was used as an *in vitro* AD model. We then assessed the potential enhancement of neurogenesis by EEA and HEEA using murine *ex vivo* neurospheres. We also administered EEA or HEEA to senescence-accelerated mouse-prone 8 (SAMP8) mice, a non-transgenic strain with accelerated aging and AD-like memory loss for evaluation of spatial learning and memory using the Morris water maze test. Finally, we performed immunohistochemical analysis for assessment of neurogenesis in mice administered EEA. Pretreatment of SH-SY5Y cells with EEA or the squalene-rich fraction of EEA, HEEA, ameliorated amyloid-β-induced cytotoxicity. Interestingly, only EEA-treated cells showed a significant increase in cell metabolism and intracellular adenosine triphosphate production. Moreover, EEA treatment significantly increased the number of neurospheres, whereas HEEA treatment significantly increased the number of β-III-tubulin+ young neurons and GFAP+ astrocytes. SAMP8 mice were given 50 mg/kg EEA or HEEA orally for 30 days. EEA and HEEA decreased escape latency in the Morris water maze in SAMP8 mice, indicating improved memory. To detect stem cells and newborn neurons, we administered BrdU for 9 days and measured BrdU+ cells in the dentate gyrus, a neurogenic stem cell niche of the hippocampus. In SAMP8 mice, EEA rapidly and significantly increased the number of BrdU+GFAP+ stem cells and their progeny, BrdU+NeuN+ mature neurons. In conclusion, our data in aggregate indicate that EEA and its constituents could be developed into a nutraceutical for promoting brain health and function against several age-related diseases, particularly AD.

## Introduction

Alzheimer's disease (AD), a growing worldwide health problem, is the most common form of dementia representing ~70% of all dementia cases. Up to now, effective therapy for AD has not been established, and no promising therapies are forthcoming. However, the number of patients with neurodegenerative diseases such as AD has been increasing; therefore, many studies on the treatment and improvement of AD with various approaches have been carried out. Lifestyle components such as exercise, intellectual stimulation activities, and sleep optimization have been reported to reduce the risk of AD and slow its progression (Fratiglioni et al., 2004; Forbes et al., 2013; Xie et al., 2013). In addition, several epidemiological studies have investigated the association between nutritional supplements, such as vitamin C, vitamin E, and omega-3 fatty acids, and the risk of AD and cognitive decline (Mangialasche et al., 2013; Shinto et al., 2014). Also, exposure to environmental enrichment has a protective effect by slowing the progression of AD and reducing AD-like cognitive impairment (Barak et al., 2013). Importantly, there has been growing interest in the potential for natural products such as nutraceuticals to treat or prevent AD.

It is estimated that about 72,500 algal species exist worldwide (Guiry, 2012). Algae produce a variety of bioactive secondary metabolites that include polyphenolic compounds, polysaccharides, steroids, fatty acids, carotenoids, mycosporine-like amino acids, halogenated compounds, polyketides, lectins, peptides, and their derivatives (Sathasivam et al., 2019). There is growing interest in exploring metabolites from algae, and attention has focused on exploring novel bioactive compounds with the potential for future therapeutic use (Blunt et al., 2017). A large number of biological functions have been found for algal natural products. They can have antioxidant, anti-inflammatory, anticancer, immunomodulatory, antidiabetic, antimicrobial, antiviral, and anticoagulant functions, tyrosinase inhibition, and ultraviolet-protective effects (Blunt et al., 2017).

*Aurantiochytrium* is an oleaginous microorganism in the Thraustochytriaceae family that has attracted attention because of its ability to produce high levels of polyunsaturated fatty acids and squalene. A research group at the University of Tsukuba found a high squalene-producing (198 mg/g) strain, 18W-13a, of *Aurantiochytrium* sp. from among 150 strains of thraustochytrids isolated in the Okinawa prefecture of Japan (Kaya et al., 2011). Squalene is a biosynthesized triterpene hydrocarbon and a precursor for all steroids in animals and plants. Squalene is used in the pharmaceutical and medical industries because it increases cellular and non-specific immune functions, decreases serum cholesterol levels, suppresses tumor proliferation, modulates fatty acid metabolism, and shows an adjuvant effect (Kelly, 1999; Aguilera et al., 2005; Kaya et al., 2011; Kumar et al., 2016; Bhilwade et al., 2019). Thus, *Aurantiochytrium* sp. may be useful in the search for new medicines and is considered to have great potential as a renewable source of chemical products, with squalene being of major interest. There have been few reports exploring the physiological effects of *Aurantiochytrium* sp. However, recent studies demonstrated that an ethanol extract obtained from *Aurantiochytrium* sp. (EEA) had anti-inflammatory effects on RAW264.7 cells (Takahashi et al., 2018). Moreover, EEA also showed an antidepressant-like effect *via* anti-inflammation (Sasaki et al., 2019a). However, the effects of *Aurantiochytrium* sp. on central nervous system (CNS) activity such as learning and memory or adult neurogenesis are unknown.

Senescence-accelerated mouse-prone 8 (SAMP8) mice are a good animal model for age-associated diseases, such as AD (Morley et al., 2012a,b). They exhibit deterioration in memory and learning accompanied by CNS inflammation, vascular impairment, gliosis, increased oxidative stress, amyloid-β (Aβ) accumulation, and tau hyperphosphorylation (Morley et al., 2012a; Cheng et al., 2014). Moreover, Griñan-Ferré et al. (2016) used environmental enrichment in SAMP8 mice and found altered chromatin-modifying gene expression in the hippocampus, increased expression of antioxidant genes, and reduced expression of pro-inflammatory genes. Another interesting study showed that environmental enrichment increased cognition, reduced Tau hyperphosphorylation, and increased synaptic protein expression in the 5XFAD mouse model of AD (Griñán-Ferré et al., 2018). Thus, not only diet but other lifestyle changes could also influence AD symptoms in humans. We previously showed that SAMP8 mice given the polyphenol 3,4,5-tricaffeoylquinic acid exhibited increased neurogenesis (Sasaki et al., 2019b). In physiologic conditions, neurogenesis occurs in the adult brain in two principal regions, the subgranular zone (SGZ) of the hippocampus (Altman and Das, 1965; Kempermann, 2002) and the wall of the lateral ventricles, the subventricular zone (SVZ) (Alvarez-Buylla and Garcia-Verdugo, 2002).

Thus, in the present study, we studied the effects of EEA on cell viability, adenosine triphosphate (ATP) production, and neural differentiation in SH-SY5Y human neuroblastoma cells. We performed complementary *in vitro* murine neurosphere assays to determine the effects of EEA on proliferation and differentiation of neural stem cells and neural precursor cells (NPCs). The SGZ has been implicated in learning and memory, and therefore, we examined learning and memory with the Morris water maze in SAMP8 mice administered EEA. Finally, we studied the effects of EEA on SGZ and SVZ stem cell activation and neurogenesis *in vivo*.

## Materials and Methods

### Preparation of Ethanol Extract of *Aurantiochytrium* sp.

Dried powders of Aurantiochytrium sp. cells were provided by Professor Makoto Watanabe (Algae Biomass and Energy System R&D Center, University of Tsukuba, Japan). The algae dried powders were stored at −80°C until extraction for maintenance of the quality. The algae dried powders were extracted using 99.5% ethanol, in the dark, and at room temperature for 2 weeks, with shaking of the mixture occurring at least once a day. At the end of the procedure, the liquid fraction was collected and filtered through a 0.22-μm filter. For both *in vitro* and *in vivo* experiments, the EEA liquid fraction was concentrated using a SpeedVac (Thermo Fisher Scientific, Japan). For *in vitro* experiments, the concentrated EEA was dissolved in serum-free Eagle's minimum essential medium (OPTI-MEM; Gibco, Japan) with sonication, and for *in vivo* experiments, the concentrated 150-mg EEA was dissolved in 10-ml milliQ water with sonication. For both *in vitro* and *in vivo* experiments, EEA was aliquoted and stored at −80°C for maintenance of quality.

### Preparation of n-Hexane Layer of Ethanol Extract of *Aurantiochytrium* Using Liquid–Liquid Distribution

To obtain the extract, *Aurantiochytrium* sp. 18W-13a strain was extracted as mentioned earlier with 99.5% EtOH for 2 weeks, and the EtOH extract was filtered and evaporated *in vacuo*. The concentrated EEA (200 mg) was dissolved in 100-ml n-Hexane, and 100-ml 90% MeOH was added to the extract. The extract was partitioned between the 90% MeOH layer and the n-Hexane layer due to the difference in their solubility. Partitioning the 90% MeOH layer and n-Hexane layer was repeated twice with 100-ml n-Hexane. Next, the n-Hexane layer containing EEA (HEEA) was concentrated using vacuo; 100 mg of this concentrate was dissolved in 1 ml of 99.5% EtOH and used for *in vitro* experiments. After dissolving in 99.5% EtOH, HEEA was aliquoted and stored at −80°C. For animal dosing for the *in vivo* assay, the HEEA was concentrated, and the dried 150-mg HEEA was dissolved in 10-ml milliQ water with sonication. The HEEA solution, for *in vivo* experiments, was also aliquoted and stored at −80°C until use for maintenance of quality.

### Preparation of Squalene

Squalene was purchased from Wako Co, Ltd. (Tokyo, Japan). For *in vitro* assays, squalene was dissolved in medium and sonicated before use in the experiment because it was difficult to dissolve in the medium.

### SH-SY5Y Cell Culture

The human neuroblastoma SH-SY5Y cell line was purchased from the American Type Culture Collection. SH-SY5Y cells were cultured in a 1:1 (v/v) mixture of Dulbecco's modified Eagle medium and Ham's F-12 medium (Gibco, Japan) supplemented with 15% heat-inactivated fetal bovine serum (Bio West, U.S.A) and 1% penicillin (5,000 μg/ml)–streptomycin (5,000 IU/ml) (Lonza, Japan) at 37°C in a humidified atmosphere of 5% CO_2_ in the air. SH-SY5Y cells were cultured in 100-mm Petri dishes or 96-well plates. OPTI-MEM was used to culture the cells for the cell viability assay.

### 3-(4,5-Dimethylthiazol-2-yl)-2,5-Diphenyltetrazolium Bromide Assay

Cell viability and mitochondrial activity were determined using a 3-(4,5-dimethylthiazol-2-yl)-2,5-diphenyltetrazolium bromide (MTT) assay to check for effects of EEA (20 μg/ml), HEEA (20 μg/ml), squalene (50 μM), and Aβ (15 μM) on cytotoxicity. SH-SY5Y cells were seeded at 2 × 10^5^ cells/ml in 96-well plates and incubated for 24 h. After 24-h incubation, SH-SY5Y cells were treated with EEA, HEEA, squalene, or Aβ for 72 h. To evaluate the neuroprotective effects of EEA, HEEA, and squalene against Aβ-induced cytotoxicity, SH-SY5Y cells were pretreated with 20 μg/ml EEA, 20 μg/ml HEEA, and 50-μM squalene for 10 min before 15-μM Aβ treatment. After sample treatment, a solution of 5 mg/ml MTT dissolved in phosphate-buffer saline (PBS) was added (10 μl/well) and incubated for another 24 h. The resulting MTT formazan was dissolved in 100 μl of 10% sodium dodecyl sulfate (w/v), and the absorbance was measured using a microtiter plate reader (Dainippon Sumitomo Pharma Co., Ltd., Japan).

### Adenosine Triphosphate Assay

The effect of EEA on ATP production of SH-SY5Y cells was determined using a luciferase luminescence assay kit (ATP reagents for cell: TOYO Ink, Tokyo, Japan). SH-SY5Y cells were seeded at 2 × 10^5^ cells/ml and incubated for 24 h. After incubation, SH-SY5Y cells were treated with 20 μg/ml EEA. After 6-, 12-, and 24-h incubation, the ATP assay reagent was added (100 μl/well) and incubated for 10 min at room temperature while avoiding light exposure. After the incubation, the solution was transferred into a white clear-bottom 96-well-plate (BD Falcon), and the luminescence was detected using a microplate reader (Dainippon Sumitomo Pharma Co., Ltd., Japan).

### Primary Neurosphere Culture

NPCs were obtained from the SVZ of 7-day-old postnatal mice following the procedure described by Torroglosa et al. (2007) . Six CD1 mice were used for each independent culture. Neurosphere cultures were maintained in a defined medium composed of Dulbecco's modified Eagle's medium/F12 medium 1:1 (v/v) with 1 mg/L gentamicin (GIBCO) and B27 supplement (Invitrogen, Carlsbad, CA). Epidermal growth factor (EGF) (20 ng/ml, GIBCO) and basic fibroblast growth factor (bFGF) (10 ng/ml, Peprotech, Frankfurt, Germany) were added to cultures to stimulate cell proliferation and culture expansion. All animal procedures were approved by the Animal Study Committee of Tsukuba University and according to the guidelines for the Care and Use of Animals approved by the Council of the Physiological Society of Japan.

To test the effect of EEA (20 μg/ml) and HEEA (20 μg/ml) on primary neurospheres, single cells from mechanically disaggregated neurospheres were seeded in anti-adherent 96 well-plates (Corning, NY, USA) at a density of 20,000 cells/ml. EEA (20 μg/ml) or HEEA (20 μg/ml), EGF (20 ng/ml), and bFGF (10 ng/ml) were added at the time of seeding. Seventy-two hours after seeding, the number of newly formed neurospheres was counted with phase microscopy. To measure neurosphere size, images of at least 50 neurospheres per well were taken. The size was measured using ImageJ software. Each treatment was performed in triplicate and repeated at least three independent times.

### Immunocytochemistry

Cells in medium without growth factors were added onto poly-L-ornithine coated 8-well-glass slide chambers (Lab-Tek) with EEA. After 72 h, cells were fixed with 4% (w/v) paraformaldehyde. After three washes, cells were incubated with a blocking solution composed of PBS containing 2.5% (w/v) bovine serum albumin for 1 h to avoid non-specific antibody binding. Primary antibody incubations were carried out overnight at 4°C in blocking solution. Then, cells were washed with PBS and incubated with the appropriate secondary antibody for 1 h.

The primary antibodies used were mouse anti-β-III-tubulin (Promega, 1:1,000) and rabbit anti-GFAP (DAKO, 1:3,000). The secondary antibodies used were donkey anti-rabbit Alexa Fluor 488 and donkey anti-mouse 594 (Invitrogen, 1:1,000). Nuclei were counterstained with 4',6-diamidino-2-phenylindole using drops of ProLong Gold Antifade Mountant (Thermo SCIENTIFIC, Japan). Fluorescence was detected with a Leica DMI 4000B epifluorescent microscope (Leica, Germany). Quantification was performed in 12 predetermined visual fields/well and 3 wells/condition. Experiments were repeated a minimum of three times, and results were expressed as the mean ± SEM.

### Animals

Adult male SAMP8 (4 months old) and senescence-accelerated resistant mouse 1 (SAMR1) (4 months old) (SLC Japan) were used for *in vivo* experiments. After acclimatization to laboratory conditions (7 days), SAMP8 mice were divided into three groups: SAMP8 control group, orally administered only with water (*n* = 10), EEA-treated group (*n* = 10), and HEEA-treated group (*n* = 7). SAMR1 mice (*n* = 10) were used as normal aging controls. Animals were housed under controlled conditions of temperature (21–23°C) and light:dark cycle (12:12 h) with free access to food and water. All animal procedures were approved by the Animal Study Committee of Tsukuba University (No. 16-041) and according to the guidelines for the Care and Use of Animals approved by the Council of the Physiological Society of Japan.

In our previous study, we orally administered 100 mg/kg of EEA for 14 consecutive days to depression model mice to evaluate its antidepressant-like activities (Sasaki et al., 2019a). Considering this previous study, 50 mg/kg was determined as the concentration for oral administration of EEA and HEEA. Oral administration (gavage) of EEA (50 mg/kg) and HEEA (50 mg/kg), dissolved in water, was performed 1 × per day for 30 days. An equal volume of water was administered to the water-administration group. In addition, BrdU (1 mg/ml), which is incorporated into proliferative cells at the S-phase of the cell cycle, was provided in the drinking water and provided to the SAMR1 control mice, the SAMP8 control group, and the EEA-treated group during nine consecutive days starting with the 14th day of oral administration.

### Morris Water Maze

Spatial learning and memory were analyzed using the Morris water maze (MWM) as previously described (Sasaki et al., 2019b). A circular pool (120 cm in diameter and 45 cm in height) was filled to a depth of 30 cm with water (23 ± 2°C) and was divided into four quadrants designated as north, east, west, and south. A platform (10 cm in diameter) was placed in the northeast quadrant and was submerged 1 cm below the water surface so that it was invisible to mice at water level. Each mouse had daily sessions of four trials for seven consecutive days using a single hidden platform in one quadrant, with the start point rotating around the other three quadrants. When the mice succeeded, they were allowed to stay for 15 s on the platform. When mice failed for more than 60 s, the experimenter assisted them in finding the platform. A probe trial was performed 24 h after the last training session of MWM. The platform was removed from the pool in this trial, and mice were allowed to swim freely for 60 s. The number of crossings over the previous position of the platform and the time spent in the target quadrant in which the platform was hidden during the acquisition trials were recorded as measures for spatial memory.

### Tissue Processing and Immunohistochemistry

Mice were killed by cervical dislocation after the MWM test; brains were removed and fixed with 4% paraformaldehyde for 24 h at 4°C. After, brains were cryoprotected in 30% sucrose (w/v) in PBS for 48 h at 4°C. Serial 30-μm coronal brain sections were obtained using a microtome on dry ice. Sections were stored at −20°C in cryoprotectant solution (ethylene glycol, glycerol, 0.1-M phosphate buffer, pH 7.4, 1:1:1:2 by volume). Pretreatment of tissue was required for BrdU detection. BrdU antigen retrieval was achieved with 1-M HCl at 38.5°C for 1 h. Sections were blocked with a solution composed of PBS, 0.1% Triton X-100, and 1% bovine serum albumin (Sigma) for 1 h, after washing abundantly with PBS. Primary antibodies were incubated overnight at 4°C. Sections were washed and incubated with fluorochrome-conjugated specific secondary antibodies overnight at 4°C. Primary antibodies used were sheep polyclonal anti-BrdU (1:500, Abcam), rat monoclonal anti-GFAP (1:400, Life Technologies), mouse monoclonal anti-NeuN (1:400, Millipore), and goat polyclonal anti-DCX (1:100, Santa Cruz). The secondary antibodies were conjugated to Alexa-488, −568, or −647 (Invitrogen Paisley, Renfrewshire, UK; 1:500). Sections were mounted with Prolong Antifade Kit (Molecular Probes, Eugene, USA). A Leica DMIRB microscope with a Hamamatsu C4742-95 digital camera or a Leica DMR microscope with a Leica DFC-500 digital camera was used to obtain epifluorescence images. Confocal images were obtained with a Zeiss LSM 710 laser scanning confocal microscope using the Z-stack and tile functions as appropriate.

Double labeling was carefully examined using a 40 × objective and the digital zoom options of FIJI (ImageJ) software. Stereological methods were used for cell counting and to estimate the number of positive cells for each specific marker using the procedures in Geribaldi-Doldán et al. (2018). Five to six animals were used per condition to analyze SVZ and SGZ neurogenesis. Cells were counted in every fifth 30-μm thick-serial coronal section. In each section, cells were counted in the lateral wall of lateral ventricles in the case of the SVZ and the SGZ in the case of the dentate gyrus (DG). Sections were analyzed under confocal microscopy at 40 × magnification. Confocal images of each section were obtained using a Zeiss LSM 710 microscope using the Z-stack function using 5–7 2-micron increments. Double labeling of individual cells was confirmed with orthogonal views of the cells. The total number of cells was determined within the volume analyzed. Mice were coded depending on the treatment, and quantification was done in a blinded analysis.

### Statistics

All analysis was performed by experimenters blind to the condition on coded slides as previously described (Kandasamy et al., 2015). Data from quantifications were collated into Microsoft Excel, and statistical analyses were performed using GraphPad Prism 6 and SPSS Statistics 22. First, to test for normality, we performed the Shapiro–Wilk test. Homogeneity of variance was confirmed with Levene's test. Accordingly, a normal distribution parametric test was used to compare means. When more than two treatment groups were compared, statistical analyses were performed using one-way ANOVA followed by Bonferroni's *post hoc* test or the Ryan–Einot–Gabriel–Welsch multiple range test. To compare two groups, Student's *t*-tests were performed. If data were not normally distributed or the variances were not homogeneous, non-parametric tests were performed to compare means using Kruskal–Wallis tests and Dunn's *post hoc* analysis. Results are expressed as mean ± SEM. Results were considered statistically significant when *p* < 0.05 for specific *p*-values.

## Results

### Ethanol Extract of *Aurantiochytrium*, n-Hexane Layer Containing Ethanol Extract of *Aurantiochytrium*, and Squalene Inhibit Amyloid-β-Induced Cell Death and Ethanol Extract of *Aurantiochytrium* Increased Adenosine Triphosphate Production

**Figure 1:**
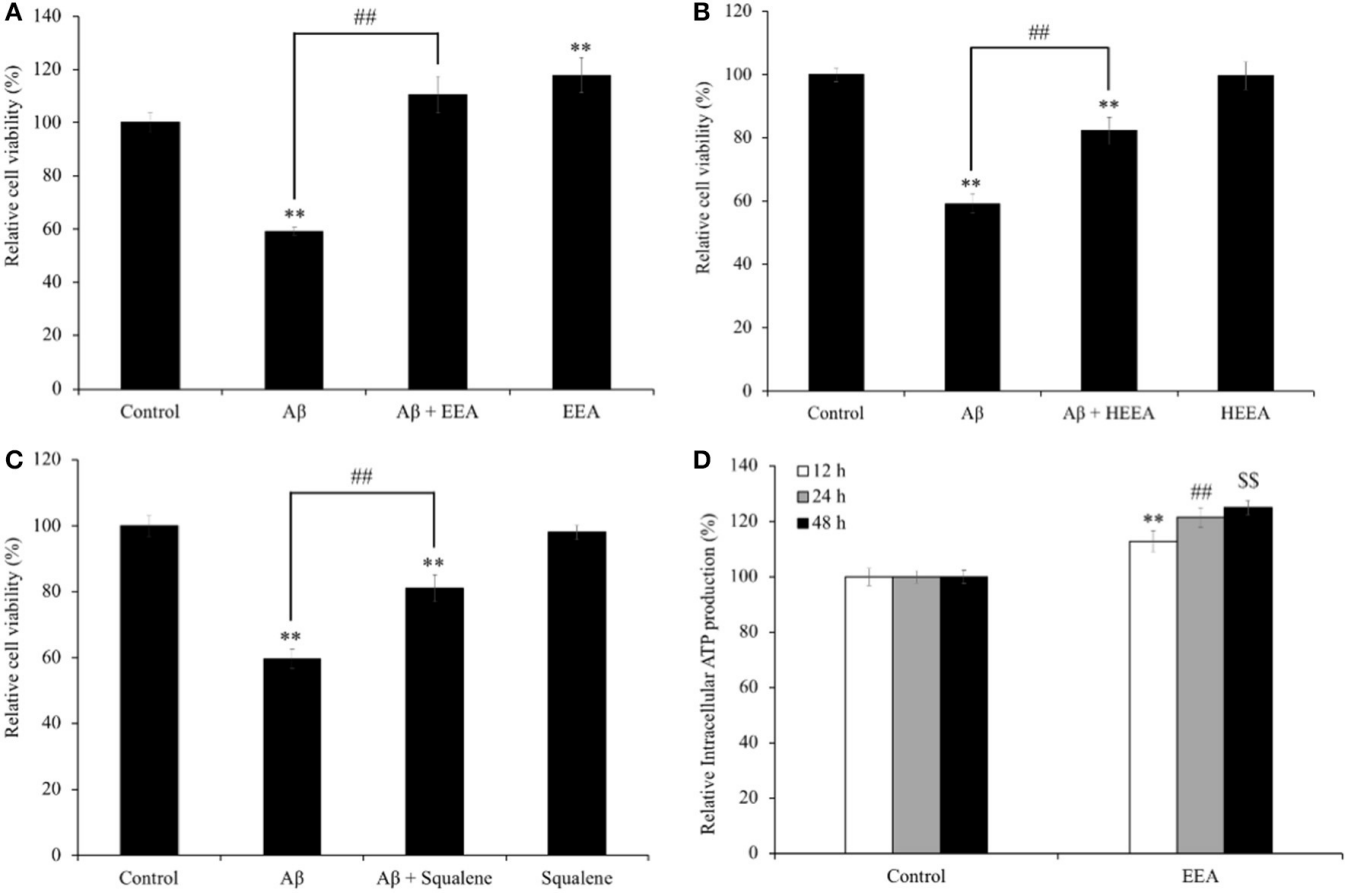
Effect of **(A)** ethanol extract of *Aurantiochytrium* sp. (EEA), **(B)** hexane layer of EEA (HEEA), and **(C)** squalene on the cell viability and Amyloid-β (Aβ)-induced changes in SH-SY5Y cells viability. **(D)** Effects of ethanol extract of EEA on ATP production of SH-SY5Y cells. In MTT assay, cells were pretreated with EEA (20 μg/ml) or HEEA (20 μg/ml) or squalene (50 μM) for 10 min, and then, cells were treated with 15-μM Aβ for 72 h. For ATP assay, cells were treated with EEA (20 μg/ml) for 6, 12, and 24 h. After treatment, intracellular ATP production level was measured. Each bar represents mean ± SEM (*n* = 5 independent experiments). **P < 0.01 *vs*. control cells, ^*##*^*P* < 0.01 *vs*. Aβ-treated cells.

SH-SY5Y cells were treated with EEA (20 μg/ml), HEEA (20 μg/ml), or squalene (20 μg/ml) for 72 h and cell viability measured with the MTT assay. To evaluate the neuroprotective effect of EEA and HEEA, SH-SY5Y cells were pretreated with 20 μg/ml EEA or 20 μg/ml HEEA for 10 min. Also, Aβ was added (final concentration: 15 μM) and co-treated with EEA or HEEA for 72 h. Then, cell viability was measured with the MTT assay. The Aβ-treated group showed a significant reduction in cell viability compared with the non-treated group (40.9 ± 1.4%, *P* < 0.01). In contrast, pretreatment with 20 μg/ml EEA or 20 μg/ml HEEA ameliorated Aβ-induced cytotoxicity (186.8 ± 6.9% and 138.9 ± 4.2% compared with 100% in Aβ-treated cells, respectively, *P* < 0.01; Figures 1A,B). Interestingly, EEA only-treated cells showed a significant increase in cell viability compared with non-treated cells (Figure 1A). Moreover, pretreatment with 50-μM squalene for 10 min also reversed Aβ-induced cell death, resulting in a significant increase in cell viability (136.0 ± 4.0% compared with 100% in Aβ-treated cells, *P* < 0.01; Figure 1C).

Because EEA increased cell viability in SH-SY5Y cells (Figure 1A), we asked if EEA affects intracellular ATP levels. In addition, Aβ is used as an oxidative stress inducer, which can decrease intracellular ATP levels by causing dysfunction in mitochondria. We found that 20 μg/ml EEA treatment significantly increased intracellular ATP levels, 112.8 ± 3.8, 121.5 ± 3.4, and 124.9 ± 2.5% compared with non-treated cells after 6-, 12-, and 24-h incubation, respectively (*P* < 0.01; Figure 1D).

### Ethanol Extract of *Aurantiochytrium* Increased Number of Neurospheres

**Figure 2:**
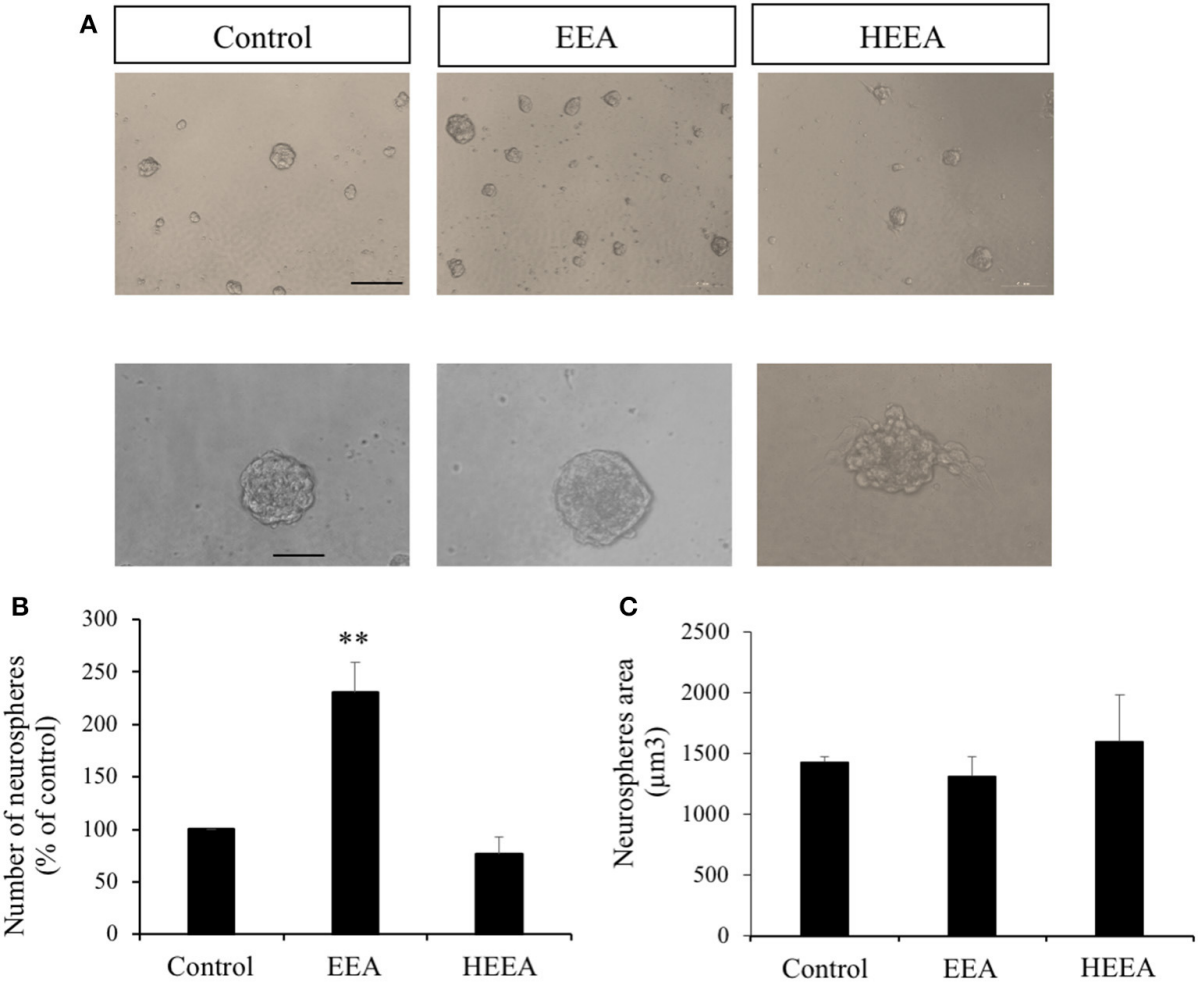
Effect of ethanol extract of *Aurantiochytrium* sp. (EEA) and hexane layer of EEA (HEEA) on NPCs proliferation. Proliferation was tested using neurosphere cultures in the presence of EGF (20 ng/ml) and bFGF (10 ng/ml). **(A)** Phase-contrast microscopy images of neurospheres treated with or without EEA (20 μg/ml) or HEEA (20 μg/ml) during 72 h. Scale bar indicates 100 μm. **(B)** Neurosphere number after treatment with grape extract. **(C)** Size of neurospheres after treatment with EEA or HEEA. **P < 0.01 compared with control in a one-way ANOVA followed by Ryan–Einot–Gabriel–Welsch multiple range test.

Based on our results that EEA increased cell viability and ATP production in SH-SY5Y cells, we evaluated the effects of EEA and HEEA on proliferation and differentiation using neurosphere assays. Murine neurospheres were treated for 72 h with 20 μg/ml EEA or 20 μg/ml HEEA. EEA treatment significantly increased the number of neurospheres (*P* < 0.01) but did not modify neurosphere size (Figure 2). In contrast to EEA, HEEA treatment did not modify neurosphere number or neurosphere size (Figures 2B,C).

### Ethanol Extract of *Aurantiochytrium* and n-Hexane Layer Containing Ethanol Extract of *Aurantiochytrium* Had Opposite Effects on Neural Differentiation

**Figure 3:**
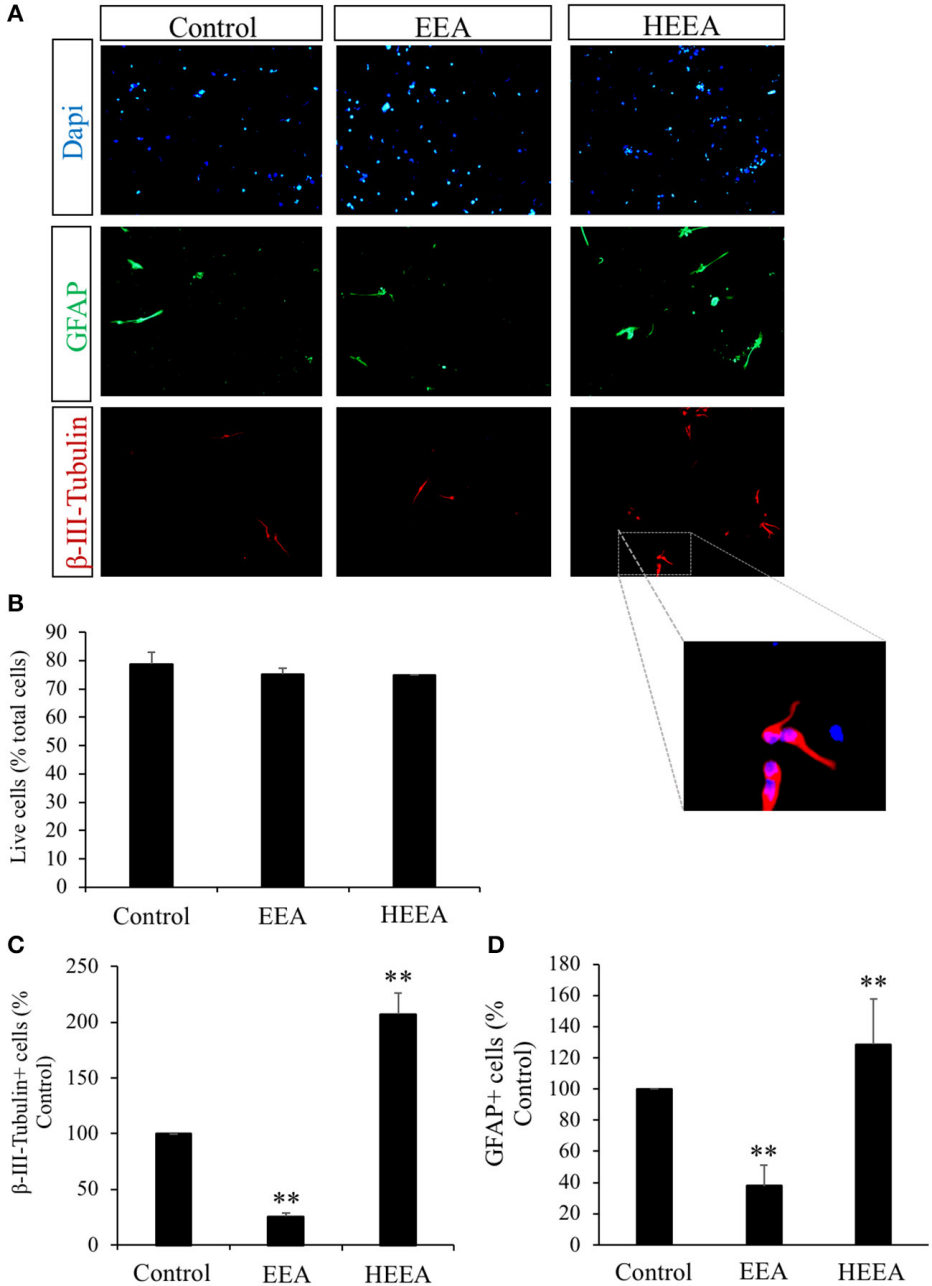
Effect of ethanol extract of *Aurantiochytrium* sp. (EEA) and hexane layer of EEA (HEEA) on NPCs proliferation. Two types of differentiation markers (β-III-tubulin for neurons and GFAP for astrocytes) were used. Neurospheres were treated with or without EEA (20 μg/ml) or HEEA (20 μg/ml) for 72 h. **(A)** Immunofluorescence images demonstrating expression of β-III-tubulin and GFAP. **(B)** Numbers of live cells after EEA or HEEA treatment. **(C)** Numbers of β-III-tubulin+ cells after treatment with EEA or HEEA. **(D)** Numbers of GFAP+ cells after treatment with EEA or HEEA. **P < 0.01 *vs*. control cells.

To study the fate of neurosphere-derived cells treated with EEA and HEEA (Figure 3A), we performed immunocytochemistry for the young neuron marker β-III-tubulin and the astrocytic and stem cell marker GFAP. Cells were seeded onto poly-L-ornithine pretreated 8-well-glass slides and cultured in media containing 20 μg/ml EEA or 20 μg/ml HEEA for 72 h. None of the treatments decreased cell viability (Figure 3B). However, EEA significantly (*P* < 0.01) decreased the number of β-III-tubulin+ cells and also the number of GFAP+ cells compared with controls, suggesting that EEA can preserve the undifferentiated state of these cells (Figures 3C,D). In contrast, treatment with HEEA increased the number of β-III-tubulin+ and GFAP+ cells compared with control and with EEA, suggesting that the squalene-rich fraction of EEA increased differentiation of these cells (Figures 3C,D).

### Ethanol Extract of *Aurantiochytrium* and n-Hexane Layer Containing Ethanol Extract of *Aurantiochytrium* Improved Spatial Learning and Memory in Senescence-Accelerated Mouse-Prone 8 Mice

**Figure 4:**
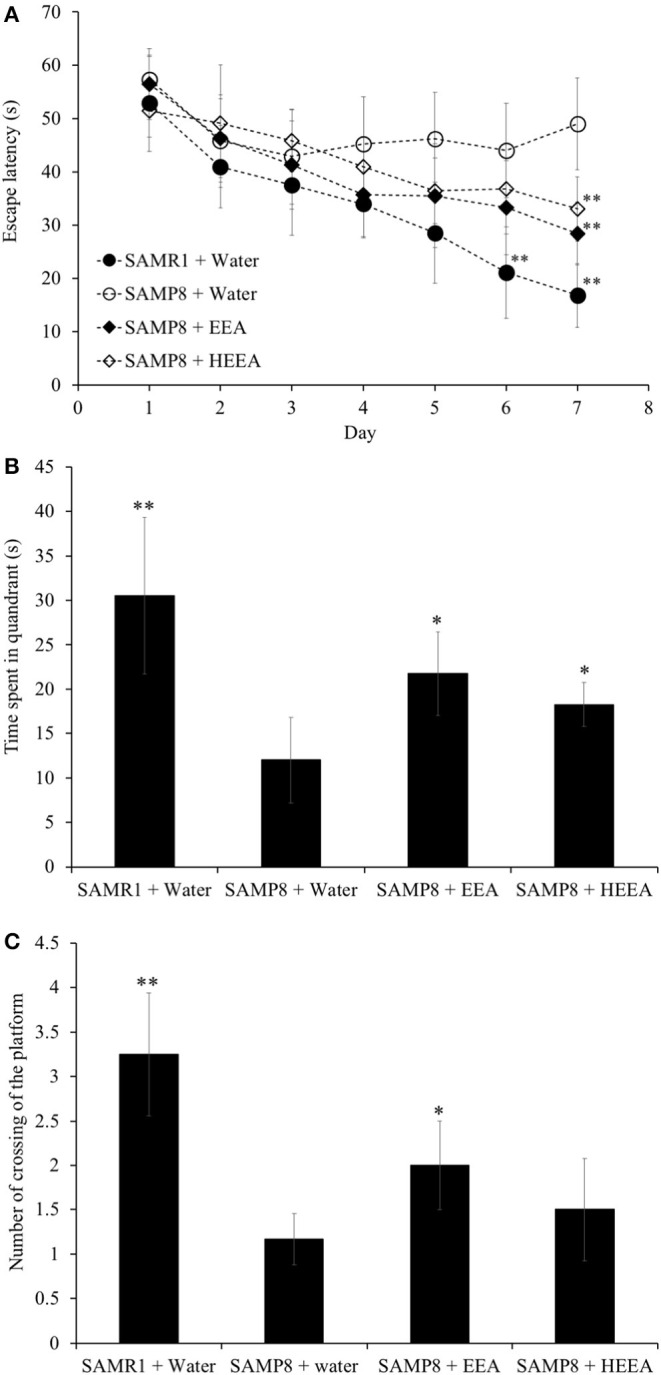
Effect of ethanol extract of *Aurantiochytrium* sp. (EEA) and hexane layer of EEA (HEEA) on the spatial learning and memory as determined by escape latency of senescence-accelerated resistant mouse 1 (SAMR1) mice, senescence-accelerated prone mouse 8 (SAMP8) mice, SAMP8 EEA-treated group, and SAMP8 HEEA-treated group determined by Morris water maze test **(A)**. Effect of EEA or HEEA on time spent in the target quadrant **(B)**. Effect of EEA or HEEA on the number of crossings of platform by SAMR1-untreated and SAMP8-treated or -untreated mice **(C)**. *P < 0.05, **P < 0.01 compared with SAMP8 + water group.

**Figure 5:**
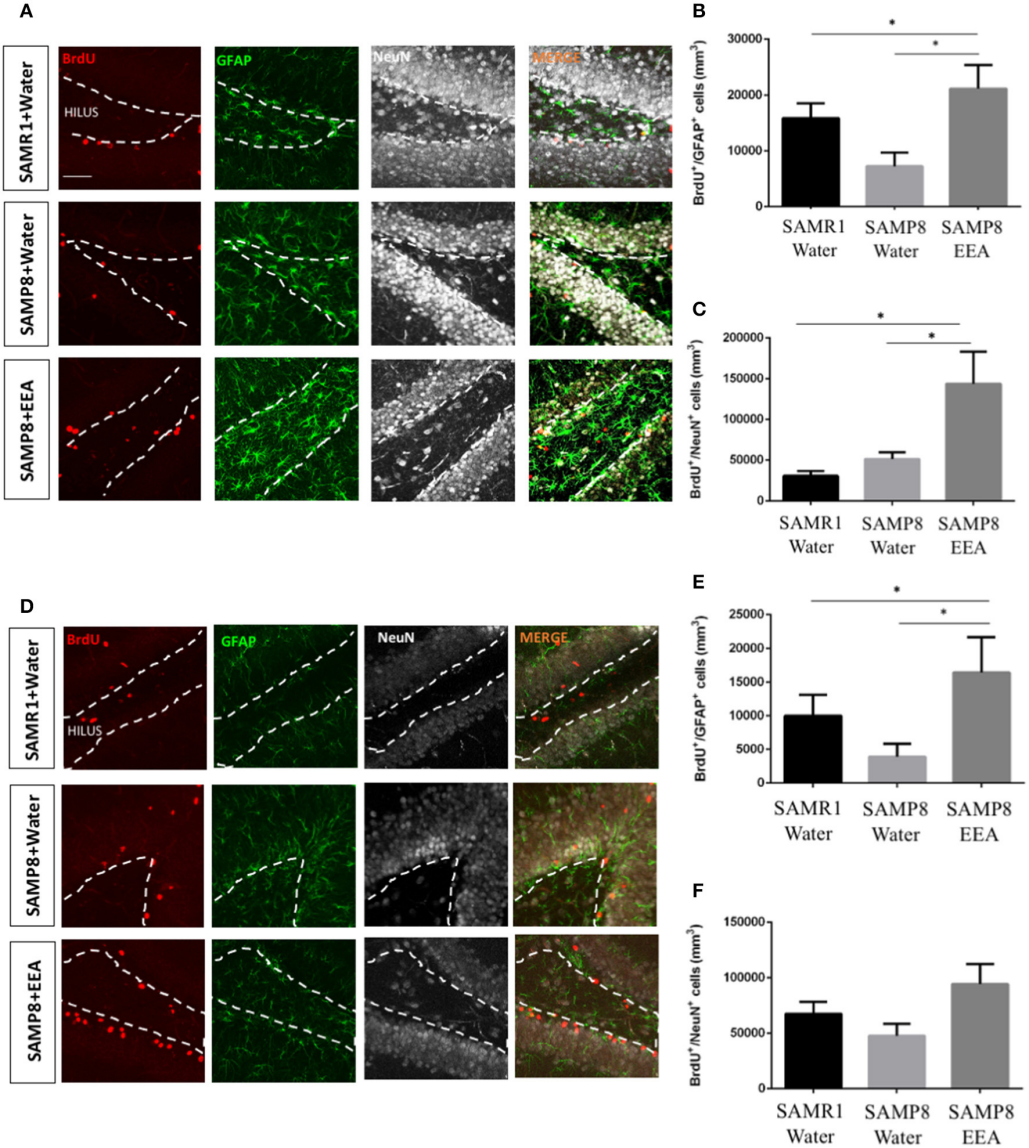
Effect of oral administration of ethanol extract of *Aurantiochytrium* sp. (EEA) on anterior and posterior dentate gyrus (DG) stem cell activation and neurogenesis. SAMP8 mice were orally administered with EEA (50 mg/kg) for 30 days. Photomicrographs show coronal sections containing the anterior **(A)** and posterior **(D)** DG processed for immunohistochemical detection of BrdU+ cells (red) and stem cell marker GFAP (green). Graphs represent number of BrdU+ cells that co-express stem cell marker GFAP in anterior **(B)** and posterior **(E)** DG. Graph representing number of BrdU+ cells that co-express mature neuronal marker NeuN in anterior **(C)** and posterior **(F)** DG. Each bar represents mean ± SEM. *P < 0.05 SAMP8 + EEA group compared with SAMP8 + water group or SAMR1 + water group **(B,C,E)**.

To evaluate the effect of EEA and HEEA (squalene-rich fraction of EEA) on spatial learning and memory in SAMP8 mice, the MWM test was performed. To assess the effect of EEA and HEEA on spatial learning and memory, we measured the time mice needed to swim to the platform (escape latency). As shown in **Figure 5A**, the escape latency of EEA-treated SAMP8 mice was significantly (*P* < 0.01) decreased compared with water-treated SAMP8 mice from the 6th and 7th day of training. There was no difference in the escape latency between SAMR1 mice and EEA-treated SAMP8 mice. Moreover, HEEA-treated SAMP8 mice also showed a significantly decreased escape latency time compared with water-treated SAMP8 mice at day 7 (Figure 4A).

Figures 4B,C show that the time spent in the target quadrant and the number of times the mouse crossed the platform was significantly higher in the EEA-treated SAMP8 group compared with the water-treated SAMP8 group (*P* < 0.01). However, no significant difference was observed in the number of crossings of the target quadrant between the HEEA-treated SAMP8 group and the water-treated SAMP8 group.

### Ethanol Extract of *Aurantiochytrium* Increases Neural Stem Cells and Neurogenesis in Hippocampus of Senescence-Accelerated Mouse-Prone 8 Mice

To test EEA (50 mg/kg) effects on neurogenic niches, we administered it orally to SAMP8 mice for 30 days, carried out the MWM test, and brains were collected and immunohistochemistry performed. We designated BrdU+ cells that co-express the NSC and astrocytic marker GFAP as SGZ stem cells. We also co-labeled BrdU+ cells with the mature neuronal marker NeuN and double-positive cells were considered to be neurons that were born during BrdU administration that had matured. We studied the effects of EEA on the anterior DG and the posterior DG separately because the former is associated with spatial memory and the latter more with limbic functions (Motta-Teixeira et al., 2015). The number of BrdU+GFAP+ cells was significantly increased in both the anterior and the posterior DG of EEA-treated SAMP8 mice compared with water-treated SAMP8 mice (*P* < 0.01) (Figures 5B,E). In the anterior DG, EEA also significantly increased the number of newborn neurons that had mature BrdU+NeuN+ cells (*P* < 0.01; Figure 5C). In the posterior DG, there was a trend toward an increased number of newborn neurons in the EEA-treated SAMP8 mice, but this did not reach statistical significance (Figures 5D,F).

**Figure 6:**
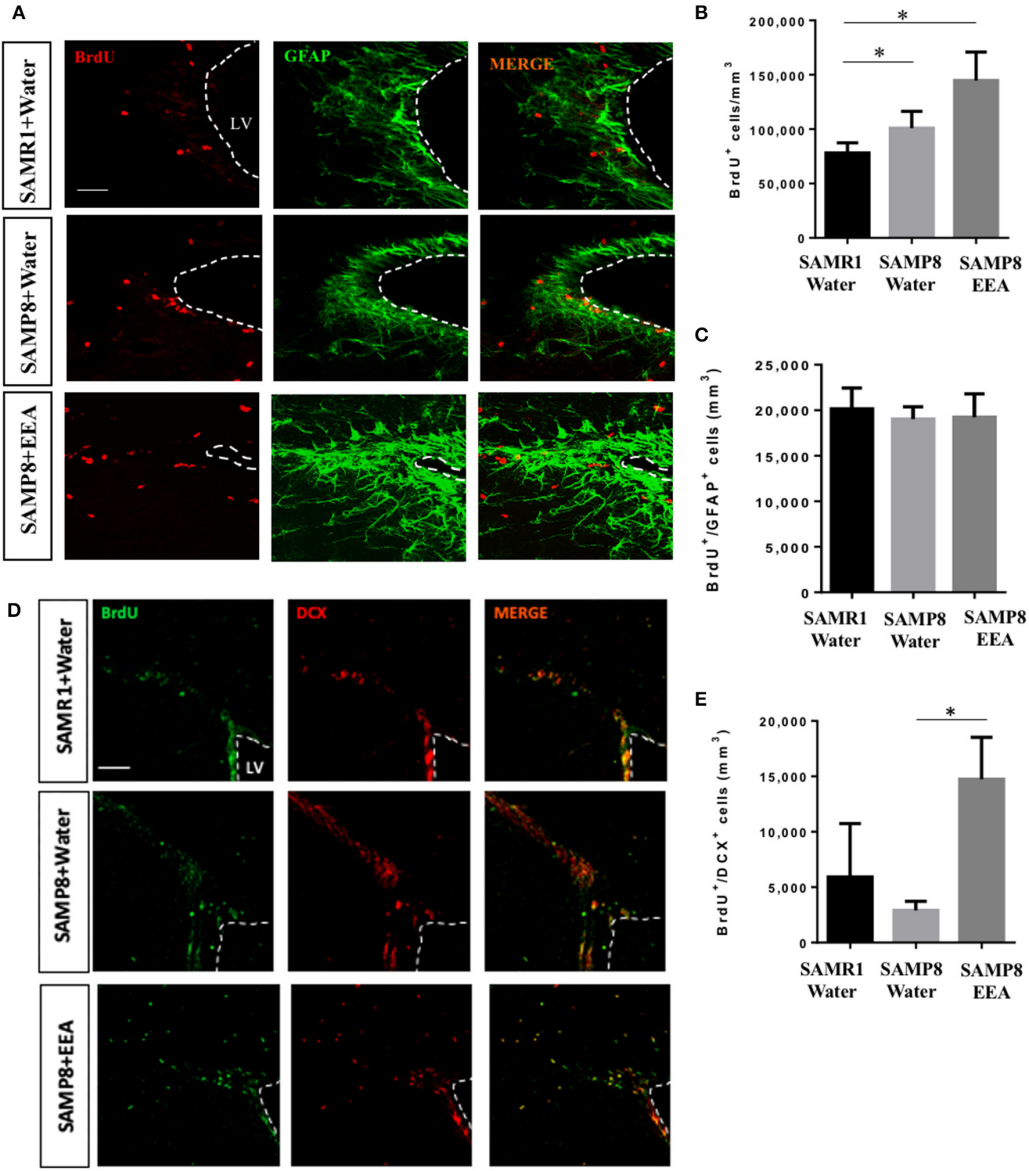
Effect of oral administration of ethanol extract of *Aurantiochytrium* sp. (EEA) on subventricular zone (SVZ) proliferation. EEA (50 mg/kg) was orally administered to SAMP8 mice for 30 days. **(A)** Photomicrograph shows SVZ in coronal sections processed for immunohistochemical detection of proliferating BrdU+ cells (red) and astrocyte marker GFAP (green), and **(D)** BrdU+ cells (green) and neuroblast marker doublecortin (DCX, red). **(B)** Graph represents number of BrdU+ cells in SVZ. **(C)** Graph showing number of BrdU cells that co-express stem cell and glial marker GFAP. **(E)** Graph showing number of BrdU cells that co-express DCX. Each bar represents mean ± SEM. *P < 0.05 SAMP8 + EEA group or SAMR1 + water group compared with SAMP8 + water group **(B,E)**.

We next asked if EEA affects the other major neurogenic niche, the SVZ. We found that the total number of BrdU+ cells was greater in SAMP8 mice compared with SAMR1 control mice, independent of treatment (Figures 6A,B). However, there were no significant differences in the number of BrdU+ cells that co-express the astrocyte and stem cell marker GFAP (Figure 6C). Nevertheless, SAMP8 EEA-treated mice exhibited a significant increase in the number of BrdU+ cells that co-express the neuroblast marker DCX in the SVZ compared with SAMP8 + water-treated mice (*P* < 0.01; Figures 6D,E). We used DCX instead of NeuN here because NeuN expression only occurs once SVZ neuroblasts have reached the olfactory bulbs, whereas DCX is expressed in newborn neurons in the SVZ (Brown et al., 2003).

We also performed double-labeling immunohistochemistry for BrdU and DCX in the DG. There was no statistically significant difference between groups in the anterior DG; however, we observed a clear tendency indicating an increase in the number of BrdU+DCX+ cells in EEA-treated SAMP8 mice compared with SAMR1 and SAMP8 water-treated mice (data not shown). Similar to the anterior DG, there was no statistically significant difference in the posterior DG among the three groups (data not shown).

## Discussion

Increasing neurogenesis by increasing NSCs is important to investigate as a treatment for AD. In this study, we focused on a microalgae *Aurantiochytrium* as a nutraceutical for the treatment of AD. We evaluated an EEA's neuroprotective effect on human neural SH-SY5Y cells and stem cell properties in murine neurosphere primary cultures. Moreover, we also evaluated the effect of EEA on spatial learning and memory and *in vivo* neurogenesis in SAMP8 mice, a good model for aging-related brain dysfunction such as AD. In this study, we have shown that the microalgae *Aurantiochytrium* has multiple neuroprotective effects on brain health. It inhibited neuronal death caused by Aβ and increased ATP in SH-SY5Y cells. In SAMP8 mice brains, it augmented hippocampal stem cells and neurogenesis and improved spatial memory loss.

These findings are important, as the development of preventive or therapeutic drugs and functional foods for these diseases is still challenging. Nutraceuticals are at the intersection of pharmaceuticals and nutrition and render health or medical benefits, including the prevention and treatment of disease. Other studies have also demonstrated the neuroprotective effects of nutraceuticals against toxic compounds associated with neurodegenerative diseases *via* mechanisms such as modulation of energy metabolism, oxidative stress, neuroinflammation, and promotion of neurogenesis *via* growth factors and neurotrophins (Dadhania et al., 2016; Pandareesh et al., 2018).

Aβ, a 39–43 amino acid peptide, is a major component of neuritic plaques and a pathological hallmark of AD, along with neurofibrillary tangles. Aβ is formed from amyloid precursor protein upon cleavage by β- and γ-secretase. This process is the amyloidogenic processing pathway that generates Aβ_1−40_ and Aβ_1−42_, which are neurotoxic. The molecular mechanism that links Aβ to the development of neurotoxicity is poorly understood, but some evidence suggests that it is caused by oxidative stress (Castillo et al., 2019), neuroinflammation (Webers et al., 2020), and dysfunction of mitochondria (Wang et al., 2020). The current study aimed to first evaluate the EEA for its *in vitro* neuroprotective effect on human SH-SY5Y cells treated with Aβ. SH-SY5Y neural cells were selected, as they are used widely for the evaluation of neurotoxic, neuroprotective, and neuroadaptive properties of various phytoconstituents and other chemical entities (Ramkumar et al., 2017; Marmit et al., 2020). In our present study, EEA (20 μg/ml)-treated cells significantly increased cell viability against the decrease of Aβ-induced cytotoxicity. Moreover, the HEEA and squalene, a major constituent of EEA, also showed the neuroprotective effect on Aβ-treated cells. Our study also showed that treatment of EEA increased cell viability as well as intracellular ATP levels. Dysfunction of energy metabolism in the brain occurs during aging but is further exacerbated in neurodegenerative diseases and is considered to be another hallmark of neurodegeneration (Camandola and Mattson, 2017). Also, the high availability of energy is important for the CNS because neurons require ATP for many critical functions, including firing action potentials, survival, proliferation, and differentiation in stem cell niches. Therefore, our results suggested that EEA may improve mitochondrial dysfunction induced by Aβ and activated mitochondria. Regulation of the amyloidogenic processing reduces Aβ production and may ameliorate Alzheimer's pathology, which is a potential target for AD. In addition, inhibition of Aβ assembly is another potential means to slow down AD pathogenesis. In the future, therefore, multiple studies on Aβ production, amyloidogenic processing, and Aβ assembly are required for understanding the mechanism underlying the neuroprotective effect of EEA (Li et al., 2020; Patil et al., 2020).

Metabolic states influence stem cell activation and neurogenesis (Llorens-Bobadilla et al., 2015), suggesting EEA could increase these processes according to the result of MTT and ATP assay. Therefore, we first analyzed the effect of EEA and HEEA in neurosphere cultures. Neurospheres are primary cultures of the stem and progenitor cells isolated from neurogenic niches consisting of floating aggregates of cells that can be used to evaluate the effects of molecules on NPCs. The size of neurospheres is indicative of NPC proliferation, whereas neurosphere numbers are indicative of self-renewal and survival. Experiments were performed in the presence of EGF and bFGF to stimulate neurosphere formation. We isolated neural stem cells and NPCs from the brain and grew neurospheres in proliferation conditions using the growth factors EGF and bFGF to promote neurosphere formation. We measured the number and size of neurospheres, where neurosphere number indirectly indicates the number of cells that enter the cell cycle or their self-renewal capacity (Reynolds and Weiss, 1996; Reynolds and Rietze, 2005) and size indicates the rate of proliferation (Torroglosa et al., 2007). We found that EEA significantly increased the number of neurospheres but not their size, suggesting an increase in this stem cell property of self-renewal upon exposure to EEA. Not only NSCs but also transit-amplifying progenitor cells can form new neurospheres, and some data suggest quiescent stem cells are not detected in neurospheres (Pastrana et al., 2011). Thus, EEA may have had an effect on multiple different cells in our cultures. In contrast to EEA, we found that HEEA did not affect neurosphere proliferation or self-renewal. We surmise that there were constituents in EEA that induced these effects but that were absent from the HEEA, hexane-purified EEA.

We subsequently found additional differences in the effects of EEA *vs*. HEEA. In terms of neurosphere differentiation, EEA decreased the number of β-III-tubulin+ neurons, whereas HEEA increased it. The same occurred with the number of GFAP+ cells. These results suggest that HEEA can induce differentiation of NPC into both neurons or glia, whereas EEA keeps them in an undifferentiated state. Recent studies showed EEA and one of its active components, squalene, have neuroprotective effects, protecting stressed neurons from cell death (Sasaki et al., 2019a). Moreover, several genes related to chemokine signaling pathways are downregulated in EEA-treated mice and may contribute to anti-inflammatory effects (Sasaki et al., 2019a). Squalene, which is concentrated in the HEEA layer, is essential in NSCs and NPC cholesterol biosynthesis. Ablation of squalene synthase promoted apoptosis in newborn neurons and reduced brain size (Saito et al., 2009). Also, disruption of cholesterol synthesis affected the radial glia fiber scaffold necessary for the migration of newborn neurons and was related to reduced cell self-renewal (Driver et al., 2016).

In our previous work, we evaluated the neuroprotective effects of several natural compounds on Aβ-treated SH-SY5Y cells as an *in vitro* model of AD (Han et al., 2010; Sasaki et al., 2013). Interestingly, this AD model showed a strong correlation with the results of our previous behavioral experiments (MWM) using SAMP8 mice (Han et al., 2010; Sasaki et al., 2013). The present study also sought to evaluate the effects of EEA and HEEA treatment in spatial and learning memory in SAMP8, a well-characterized model for studying brain aging and neurodegeneration (Morley et al., 2012a,b) using the MSM test. The MWM is a widely used tool to assess spatial learning and memory in rodents. In the MWM, the animal finds or is placed on a platform concealed under the surface of the water in a pool. The time each mouse takes to subsequently swim to the platform indicates how quickly it can recall the location of the platform. SAMP8 mice show age-related deterioration in learning and memory ability with comprehensive brain pathological changes, including the deterioration of amyloid pathology by alterations in the amyloid pathway (Porquet et al., 2015) and have been used as a non-transgenic murine model for accelerated senescence and AD (Hong et al., 2020). In our study, we found that SAMP8 mice treated with EEA or HEEA demonstrated increased learning ability in the MWM test. Thus, our results suggested that EEA and HEEA may potentially restore neuronal damage and death induced by Aβ, which may help improve spatial learning and memory in SAMP8 mice.

Using gavage, EEA was administered to SAMP8 and SAMR1 mice to study proliferation *in vivo* in the neurogenic stem cell niches. The hippocampal SGZ has been shown to mediate learning and memory (Anacker and Hen, 2017). In the SGZ, we found significantly more cells that co-express the proliferation marker BrdU and the mature neuronal marker NeuN, indicating that EEA encourages neurogenesis in this region. Additionally, we observed increased numbers of BrdU+ cells co-expressing the glial and stem cell marker GFAP in the anterior SGZ. This increase suggests that quiescent NSCs could be activated by EEA, eventually increasing neuronal production. One potential mechanism is related to fatty acid metabolism. EEA could induce *de novo* lipogenesis, which is key in neurogenesis (Knobloch et al., 2013) and so ameliorate SAMP8 cognitive deficiencies. We also studied the other important neurogenesis niche, the SVZ, and found more BrdU+ cells that co-express the neuroblast marker DCX in the SVZ of SAMP8 mice treated with EEA. In contrast, we found no significant changes in the number of BrdU+ cells that co-express the glial and neural stem cell marker GFAP, suggesting that EEA induced neurogenesis in the SVZ but did not activate NSCs. Moreover, our hippocampal results indicate increased neurogenesis after EEA administration, and this may contribute to their enhanced performance in the MWM spatial memory test. However, it is important to point out that EEA administered mice may have altered strategy use, motivation, or stress resilience, and the MWM results may not be entirely due to increased hippocampal neurogenesis. The relationship between adult neurogenesis and AD has been recently debated (Moreno-Jiménez et al., 2019; Scopa et al., 2020). Previous studies have reported reduced expression of neurogenesis markers in the SVZ and DG regions of postmortem AD brain. Moreover, several studies demonstrated increased neurogenesis in animal models and humanized three-dimensional systems of AD (Bhattarai et al., 2016; Choi et al., 2018; Papadimitriou et al., 2018).

## Conclusion

Taken together, our present research suggests that *Aurantiochytrium* improves spatial learning and memory in SAMP8 mice, an animal model of aging. This was accompanied by enhancement of neurogenesis in the hippocampal DG. Our new findings in aggregate suggest that EEA could be used as a new therapeutic agent for the treatment of neurodegenerative diseases or other age-related problems such as AD.

## Data Availability Statement

The raw data supporting the conclusions of this article will be made available by the authors, without undue reservation.

## Ethics Statement

The animal study was reviewed and approved by the Animal Study Committee of Tsukuba University.

## Author Contributions

KS, FS, and HI conceived and designed the experiments. KS, NG-D, and QW performed the experiments. KS and NG-D prepared the figures and tables. KS and NG-D analyzed and interpreted the results and wrote the paper. FS and HI edited and revised the manuscript. All authors contributed to the article and approved the submitted version.

## Conflict of Interest

The authors declare that the research was conducted in the absence of any commercial or financial relationships that could be construed as a potential conflict of interest.
